# Anti-Osteoclastogenic Activity of Praeruptorin A via Inhibition of p38/Akt-c-Fos-NFATc1 Signaling and PLCγ-Independent Ca^2+^ Oscillation

**DOI:** 10.1371/journal.pone.0088974

**Published:** 2014-02-21

**Authors:** Jeong-Tae Yeon, Kwang-Jin Kim, Sik-Won Choi, Seong-Hee Moon, Young Sik Park, Byung Jun Ryu, Jaemin Oh, Min Seuk Kim, Munkhsoyol Erkhembaatar, Young-Jin Son, Seong Hwan Kim

**Affiliations:** 1 Research Institute of Basic Science, Sunchon National University, Suncheon, Republic of Korea; 2 Laboratory of Translational Therapeutics, Pharmacology Research Center, Division of Drug Discovery Research, Korea Research Institute of Chemical Technology, Daejeon, Republic of Korea; 3 Department of Biology, Chungnam National University, Daejeon, Republic of Korea; 4 Herbal Medicine Research Division, National Institute of Food & Drug Safety Evaluation, Cheongwon, Republic of Korea; 5 Graduate School of New Drug Discovery and Development, Chungnam National University, Daejeon, Republic of Korea; 6 Department of Anatomy & Institute for Skeletal Diseases, School of Medicine, Wongkwang University, Iksan, Republic of Korea; 7 Department of Oral Physiology, School of Dentistry, Wongkwang University, Iksan, Republic of Korea; University of California, Los Angeles, United States of America

## Abstract

**Background:**

A decrease of bone mass is a major risk factor for fracture. Several natural products have traditionally been used as herbal medicines to prevent and/or treat bone disorders including osteoporosis. Praeruptorin A is isolated from the dry root extract of *Peucedanum praeruptorum* Dunn and has several biological activities, but its anti-osteoporotic activity has not been studied yet.

**Materials and Methods:**

The effect of praeruptorin A on the differentiation of bone marrow–derived macrophages into osteoclasts was examined by phenotype assay and confirmed by real-time PCR and immunoblotting. The involvement of NFATc1 in the anti-osteoclastogenic action of praeruptorin A was evaluated by its lentiviral ectopic expression. Intracellular Ca^2+^ levels were also measured.

**Results:**

Praeruptorin A inhibited the RANKL-stimulated osteoclast differentiation accompanied by inhibition of p38 and Akt signaling, which could be the reason for praeruptorin A-downregulated expression levels of c-Fos and NFATc1, transcription factors that regulate osteoclast-specific genes, as well as osteoclast fusion-related molecules. The anti-osteoclastogenic effect of praeruptorin A was rescued by overexpression of NFATc1. Praeruptorin A strongly prevented the RANKL-induced Ca^2+^ oscillation without any changes in the phosphorylation of PLCγ.

**Conclusion:**

Praeruptorin A could exhibit its anti-osteoclastogenic activity by inhibiting p38/Akt-c-Fos-NFATc1 signaling and PLCγ-independent Ca^2+^ oscillation.

## Introduction

Bone fracture is a public health problem because it occurs easily in patients with bone-related disorders including osteoporosis. As the elderly population is rapidly increasing, the medical costs of hospitalization caused by fractures have been become a serious social issue [1], [2].

Bone homeostasis depends on the balance between osteoblastic bone formation and osteoclastic bone resorption, but an imbalance caused by an increased number of osteoclasts or overactivation can lead to impaired bone structure and low bone mass, which are common characteristics in patients with bone disorders [3], [4]. Therefore, a method to pharmaceutically inhibit osteoclast differentiation is one of the therapeutic strategies for preventing and/or treating bone disorders and related fractures [5].

Clinically, osteoclast-targeting bisphosphonates have been widely used to treat patients with osteoporosis and/or prevent osteoporotic fracture. The orally available bisphosphonates principally inhibit the activation of osteoclasts by binding to hydroxyapatite [6], but recently bisphosphonate-related side effects including acute phase response, hypocalcaemia, secondary hyperparathyroidism, upper gastrointestinal tract problems, musculoskeletal pain, rental toxicity and osteonecrosis of the jaw have been reported [7]. Therefore, there have been several basic and clinical efforts to find anti-osteoporotic phytochemicals in order to minimize adverse side effects [8]–[10].

**Figure 1:**
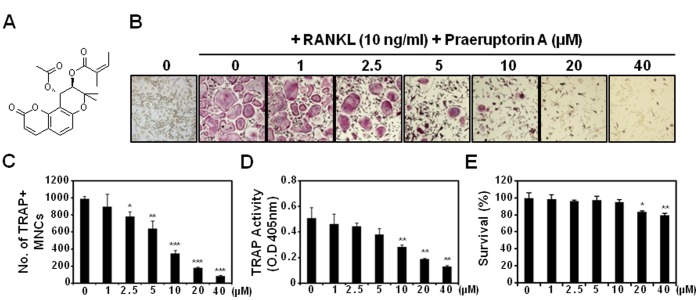
Effect of praeruptorin A on RANKL-induced osteoclast differentiation. (A) Chemical structure of praeruptorin A. (B) BMMs were pretreated with vehicle (0.1% DMSO) or praeruptorin A for 2 h and then incubated with RANKL (10 ng/ml) and M-CSF (30 ng/ml) for 4 days. Multinucleated cells were fixed, permeabilized, and stained with TRAP solution. Mature TRAP-positive multinucleated osteoclasts (MNCs) were photographed under a light microscope. TRAP-positive MNCs (nuclear number >3) were counted (C), and TRAP activity of osteoclasts was measured (D). (E) The effect of praeruptorin A on the viability of BMMs was evaluated by CCK-8 assay. *, *P*<0.05; **, *P*<0.01; ***P<0.001.

Praeruptorin A (Fig. 1A) is isolated from the dry root extract of *Peucedanum praeruptorum* Dunn, which has been used as an herbal medicine with several pharmacological activities [11]–[14]. Recently, praeruptorin A has been shown to suppress the lipopolysaccharide (LPS)-induced inflammatory response in RAW264.7 cells [15]. Since LPS is a potent stimulator of both inflammation and bone resorption [16], [17] and several phytochemicals exhibit dual anti-inflammatory and anti-resorptive activities [18], [19], in this study, the anti-osteoclastogenic activity of praeruptorin A was investigated. The functional involvement of praeruptorin A in osteoclast differentiation is not yet clearly understood.

## Materials and Methods

### Reagents

Praeruptorin A was purchased from Stanford Chemicals (CA). Mouse soluble receptor activator of nuclear factor-κB ligand (RANKL) and macrophage-colony stimulating factor (M-CSF) were purchased from R&D Systems (MN). Penicillin, streptomycin, cell culture medium, and fetal bovine serum (FBS) were purchased from Invitrogen Life Technologies (NY). The CCK-8 assay kit was from Dojindo Molecular Technologies (ML). Antibodies against c-Fos, NFATc1 and actin were from Santa Cruz Biotechnology (CA). Antibodies against p-p38, p38, p-JNK, JNK, p-ERK, ERK, Akt, p-Akt (Ser473), PLCγ and p-PLCγ were obtained from Cell Signaling Technology (MA).

### Osteoclast Differentiation

This study was carried out in strict accordance with the recommendations in the Standard Protocol for Animal Study of Korea Research Institute of Chemical Technology (KRICT; Permit No. 2012-7D-02-01). The protocol (ID No. 7D-M1) was approved by the Institutional Animal Care and Use Committee of KRICT (IACUC-KRICT). All efforts were made to minimize suffering. In detail, after cervical dislocation, bone marrow cells were obtained from 5-wk-old male ICR mice (Damool Science, Daejeon, Korea) by flushing femurs and tibias with α-MEM supplemented with antibiotics (100 units/ml penicillin and 100 µg/ml streptomycin). Bone marrow cells were cultured for 1 day on a culture dish in α-MEM supplemented with 10% FBS and M-CSF (10 ng/ml). Non-adherent bone marrow cells were plated on a Petri dish and cultured for 3 days in the presence of M-CSF (30 ng/ml). After non-adherent cells were washed out, adherent cells were used as bone marrow-derived macrophages (BMMs). For osteoclastogenesis, BMMs (1×10^4^ cells/well in a 96-well plate or 3×10^5^ cells/well in a 6-well plate) were cultured in the presence of M-CSF (30 ng/ml) and RANKL (10 ng/ml) for 4 days.

### Tartrate-resistant Acid Phosphatase (TRAP) Staining and Activity Assay

Cells were fixed with 3.7% formaldehyde for 5 min, permeabilized with 0.1% Triton X-100 for 5 min, and stained with the Leukocyte Acid Phosphatase Kit 387-A (Sigma-Aldrich, MO). TRAP-positive multinuclear cells with three or more nuclei were counted as osteoclasts. To measure TRAP activity, cells were treated with TRAP buffer (100 mM sodium citrate, pH 5.0, 50 mM sodium tartrate) including 3 mM *p*-nitrophenyl phosphate (Sigma-Aldrich) at 37°C for 5min. Reaction mixtures were transferred into a new plate containing an equal volume of 0.1 N NaOH, and optical density values were determined at 405 nm in a Wallac EnVision microplate reader (PerkinElmer, Finland).

### Cytotoxicity Assay

BMMs were plated at a density of 1 × 10^4^ cells/well on a 96-well plate in triplicate. After treatment with M-CSF (30 ng/ml) and praeruptorin A, cells were cultured for 3 days. Then, cell viability was measured with the CCK-8 kit according to the manufacturer’s protocol.

### Western Blot Analysis

Western blot analysis was performed as described previously [9], [10]. Briefly, cells were washed, lysed, and centrifuged at 10,000× *g* for 15 min. After protein quantification of the supernatants by the BCA protein assay (Pierce, IL), proteins were denatured, separated on SDS-PAGE gels, and transferred onto PVDF membranes (Millipore, CA). After incubation with antibody, the membranes were developed using SuperSignal West Femto Maximum Sensitivity Substrate (Pierce) and visualized with the LAS-3000 luminescent image analyzer (Fuji Photo Film Co., Ltd., Japan). ImageJ software-based quantification of the detected bands was performed, and the relative, normalized ratio between the density of phosphorylated form and that of the protein itself or actin was presented in each figure.

### NFATc1 Luciferase Activity Assay

Luciferase reporter plasmid, NFATc1-luc vector was purchased from Clontech (CA) and pRL-Renilla control vector was purchased from Promega (WI). The full-length human RANK cDNA was amplified from human leukocyte cDNA from Clontech (CA) and cloned into the HindIII-EcoRI site of pcDNA3.1 (Invitrogen). Recombinant human RANKL (hRNAKL) was purchased from R&D Systems (CA). For measurement of luciferase activity, human embryonic kidney HEK293T cells were plated at a density of 5×10^4^ cells/well on a 48-well plate in triplicate for 1 day. Plasmids containing NFAT-luc (100 ng/well), pcDNA3.1-RANK (100 ng/well), and pRL-Renilla (20 ng/well) were transfected into HEK293T cells with Lipofectamine 2000 (Invitrogen, CA) according to the manufacturer’s protocol. After 6 h, transfected HEK293T cells were treated with hRANKL (50 ng/mL) and praeruptorin A for 2 days. The luciferase activity was detected in cell extracts with the Dual-Luciferase Reporter Assay System (Promega, WI) and Wallac EnVision microplate reader (PerkinElmer). Luciferase activity assessed in triplicates was normalized to the average renilla luciferase activity.

### Real-time PCR

Real-time PCR was performed as described previously [9], [10]. Primers were chosen with the online Primer3 design program [20]. The primer sets used in this study are shown in Table 1. Briefly, total RNA was isolated with TRIzol reagent, and the first-strand cDNA was synthesized with the Omniscript RT kit (Qiagen) according to the manufacturer’s protocol. SYBR green-based QPCR was performed with the Stratagene Mx3000P Real-Time PCR system and Brilliant SYBR Green Master Mix (Stratagene, CA). All reactions were run in triplicate, and data were analyzed by the 2^−ΔΔC^_T_ method [21]. Glyceraldehyde-3-phosphate dehydrogenase (GAPDH) was used as an internal standard gene. The statistical significance was determined by Student’s *t*-test with GAPDH-normalized 2^−ΔΔC^_T_ values; differences were considered significant at *p*<0.05.

**Table 1:** Primer sequences used in this study.

Target Gene	Forward Primer (5′–3′)	Reverse Primer (5′–3′)
TRAP	GATGACTTTGCCAGTCAGCA	ACATAGCCCACACCGTTCTC
NFATc1	GGGTCAGTGTGACCGAAGAT	GGAAGTCAGAAGTGGGTGGA
OSCAR	AGGGAAACCTCATCCGTTTG	GAGCCGGAAATAAGGCACAG
Cathepsin K	GGCCAACTCAAGAAGAAAAC	GTGCTTGCTTCCCTTCTGG
DC-STAMP	CCAAGGAGTCGTCCATGATT	GGCTGCTTTGATCGTTTCTC
c-Src	CCAGGCTGAGGAGTGGTACT	CAGCTTGCGGATCTTGTAGT
GAPDH	AACTTTGGCATTGTGGAAGG	ACACATTGGGGGTAGGAACA

### Retrovirus Preparation and Infection

Retrovirus preparation and infection were conducted as described previously [22]. Briefly, to obtain retroviral particles, pMX-IRES-green fluorescent protein (GFP; the control) or pMX-CA-NFATc1-GFP containing constitutively active (CA)-NFATc1 was transfected into Plat-E cells (Cell Biolabs, Inc., CA) using Lipofectamine 2000 reagent according to the manufacturer’s protocol. After viral particles were collected from the culture medium for 48 h, BMMs were incubated with those in the presence of M-CSF (30 ng/ml) and polybrene (10 µg/mL) for 8 h. For the osteoclast formation assay, BMMs were treated with RANKL (10 ng/ml), M-CSF (30 ng/ml) and praeruptorin A (10 µM) for 4 days.

### Intracellular Ca^2+^ Measurement

Isolated BMMs were seeded at a density of 1×10^5^ cells on the cover glass and cultured in the presence of RANKL (10 ng/ml) and M-CSF (30 ng/ml) for 24 h. Cells were incubated with vehicle (DMSO) or praeruptorin A (10 µM) for 2 h, treated with Fura-2, AM (5 µM), and then placed in the perfusion chamber to allow continuous perfusion of bath solution (140 mM NaCl, 5 mM KCl, 1 mM MgCl_2_, 1 mM CaCl_2_, 10 mM HEPES, and 10 mM glucose, pH 7.4, 310 mOsm) until each experiment was complete. Fluorescent dyes inside the cells were excited at wavelengths of 340 nm and 380 nm sequentially and the emitted fluorescence was passed through 510 nm cut-off filter. Each image was collected with a CCD camera and analyzed with MetaFluor software. All results were digitized to the mean of the ratio (340 nm/380 nm).

### Statistical Analysis

All quantitative values are presented as mean ± SD. Statistical differences were analyzed using Student’s *t-*test. A value of *p*<0.05 was considered significant.

## Results

### Praeruptorin A Inhibits RANKL-induced Osteoclast Differentiation

The effect of praeruptorin A on RANKL-induced osteoclastogenesis was evaluated in BMMs. Pre-treatment with praeruptorin A before RANKL treatment strongly prevented the RANKL-induced formation of TRAP-positive multinucleated cells in a dose-dependent manner (Fig. 1B and C). Consistent with these results, TRAP activity was also significantly inhibited by praeruptorin A above 10 µM (Fig. 1D). To clarify the possibility that the anti-osteoclastogenic activity of praeruptorin A could be due to its cytotoxicity in BMMs, we investigated the cytotoxic effect of praeruptorin A in BMMs. As shown in Fig. 1E, praeruptorin A exhibited significant cytotoxicity above 20 µM, suggesting that anti-osteoclastogenic activity of praeruptorin A without any cytotoxicity could be expected under 10 µM. Hoechst-stained nuclei supported no difference in cell cytotoxicity or spreading by praeruptorin A at 10 µM (Fig. S1). Therefore, the cells were treated with 10 µM of praeruptorin A in subsequent experiments to evaluate its anti-osteoclastogenic mechanism. At 10 µM, praeruptorin A also strongly inhibited osteoclast formation by inhibiting cell fusion; when it was added before fusion of preosteoclasts (or treated in the differentiation day 3; Fig. S2), the formation of multinucleated osteoclasts was significantly inhibited.

### Praeruptorin A Inhibits RANKL-induced Activation of p38 and Akt

**Figure 2:**
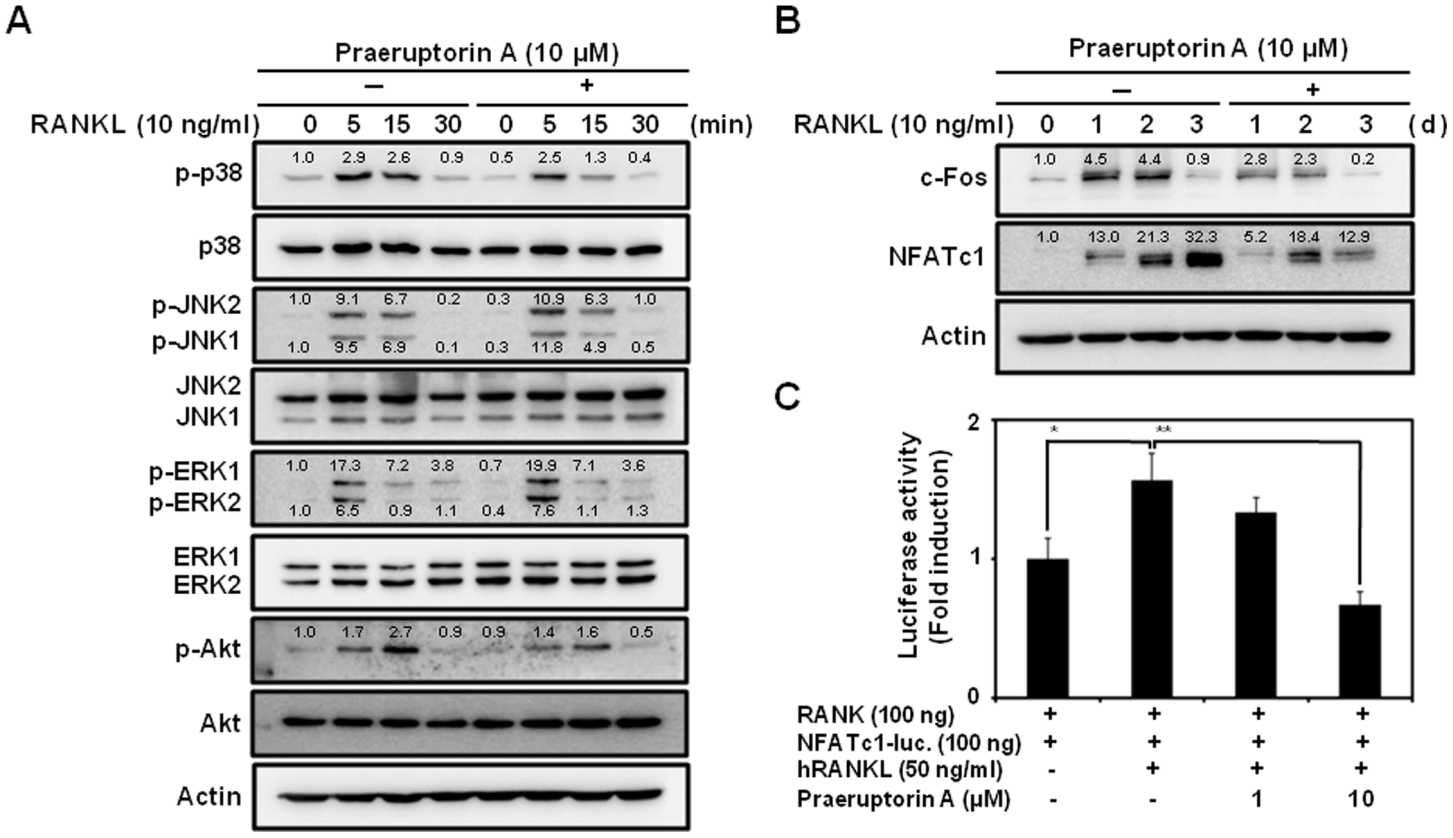
Effect of praeruptorin A on RANKL-induced activation or expression of osteoclast-specific signaling molecules and transcription factors. The effects of praeruptorin A on RANKL-induced phosphorylation of MAP kinases and Akt (A) and expression of transcription factors, c-Fos and NFATc1 (B), were evaluated by Western blot analysis. BMMs were pre-treated with praeruptorin A (10 µM) 2 h before treatment with RANKL (10 ng/ml) and M-CSF (30 ng/ml). Actin was used as an internal control. Densitometric analysis was performed using ImageJ software and the relative, normalized ratios of p-p38/p38, p-JNKs/JNKs, p-ERKs/ERK, p-Akt/Akt, c-Fos/actin and NFATc1/actin were presented. (C) The effect of praeruptorin A on the transcriptional activity of NFATc1 was evaluated by luciferase activity assay as described in ‘Materials and Methods’. *, *P*<0.05; **, *P*<0.01.

Anti-osteoclastogenic activity of praeruptorin A was confirmed by staining actin ring of mature osteoclasts (Fig. S3). This result was consistent with Fig. 1B. To elucidate the anti-osteoclastogenic mechanism of praeruptorin A, we investigated its effect on the activation of signaling molecules including JNK, p38, ERK, and Akt, which are known to play a role in the early stage of RANKL-induced osteoclast differentiation. After RANKL treatment, all signaling molecules were activated in 5–15 min, but pre-treatment with praeruptorin A 30 min before RANKL treatment attenuated the RANKL-induced phosphorylation of p38 and Akt within 15 min after RANKL treatment (Fig. 2A). The RANKL-induced activation of ERK and JNK were not changed by praeruptorin A.

### Praeruptorin A Inhibits RANKL-induced Expression of c-Fos and NFATc1

Additionally, the inhibitory effect of praeruptorin A on the expression of transcription factors such as c-Fos and NFATc1 was evaluated by Western blot analysis. As shown in Fig. 2B, RANKL induced the expression of c-Fos and NFATc1 in the early and late stages of osteoclastogenesis, respectively. However, those inductions were inhibited by praeruptorin A. In particular, on day 3, the RANKL-induced expression of NFATc1 was strongly blocked by praeruptorin A.

Additionally, the inhibitory effect of praeruptorin A on the activation of NFATc1 was revealed by the NFATc1 luciferase activity assay (Fig. 2C); RANKL significantly induced the transcriptional activity of NFATc1 in HEK293T cells transfected with RANK plasmid and NFATc1 firefly-luciferase reporter plasmid, but the addition of 10 µM praeruptorin A significantly inhibited the RANKL-induced transcriptional activity of NFATc1.

### Praeruptorin A Inhibits RANKL-induced mRNA Expression of Osteoclast-Specific Genes

**Figure 3:**
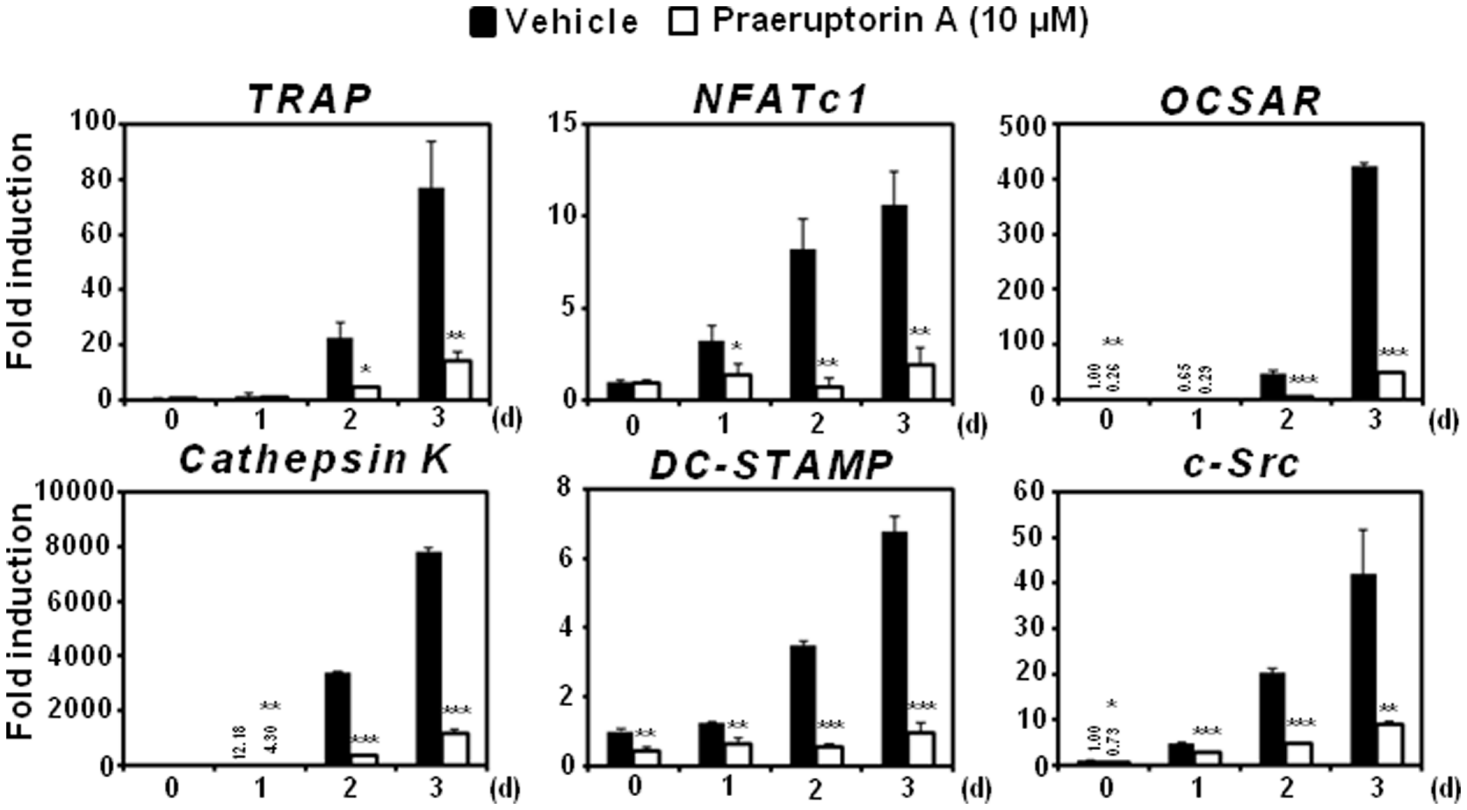
Effect of praeruptorin A on RANKL-induced mRNA expressions of osteoclastic-specific genes. BMMs were treated with vehicle (DMSO) or praeruptorin A (10 µM) for 2 h and then RANKL (10 ng/ml) was added into cells. The mRNA expression levels of osteoclastic-specific genes were analyzed by real-time PCR. *, *P*<0.05; **, *P*<0.01; ***P<0.001.

The anti-osteoclastogenic activity of praeruptorin A was confirmed by evaluating the mRNA expression levels of osteoclast-specific genes. Consistent with the previous data, praeruptorin A significantly attenuated the RANKL-induced mRNA expressions of TRAP and NFATc1 (Fig. 3). Since NFATc1 can regulate the mRNA levels of osteoclast-related molecules including osteoclast-associated receptor (OSCAR), dendrite cell-specific transmembrane protein (DC-STAMP), cathepsin K and c-Src, we further investigated whether praeruptorin A could affect the mRNA expression of these genes during osteoclastogenesis. As expected, the mRNA expressions of these molecules were highly induced by RANKL, but praeruptorin A dramatically suppressed those inductions in a dose-dependent manner.

### Ectopic NFATc1 Rescues Anti-osteoclastogenic Action of Praeruptorin A

**Figure 4:**
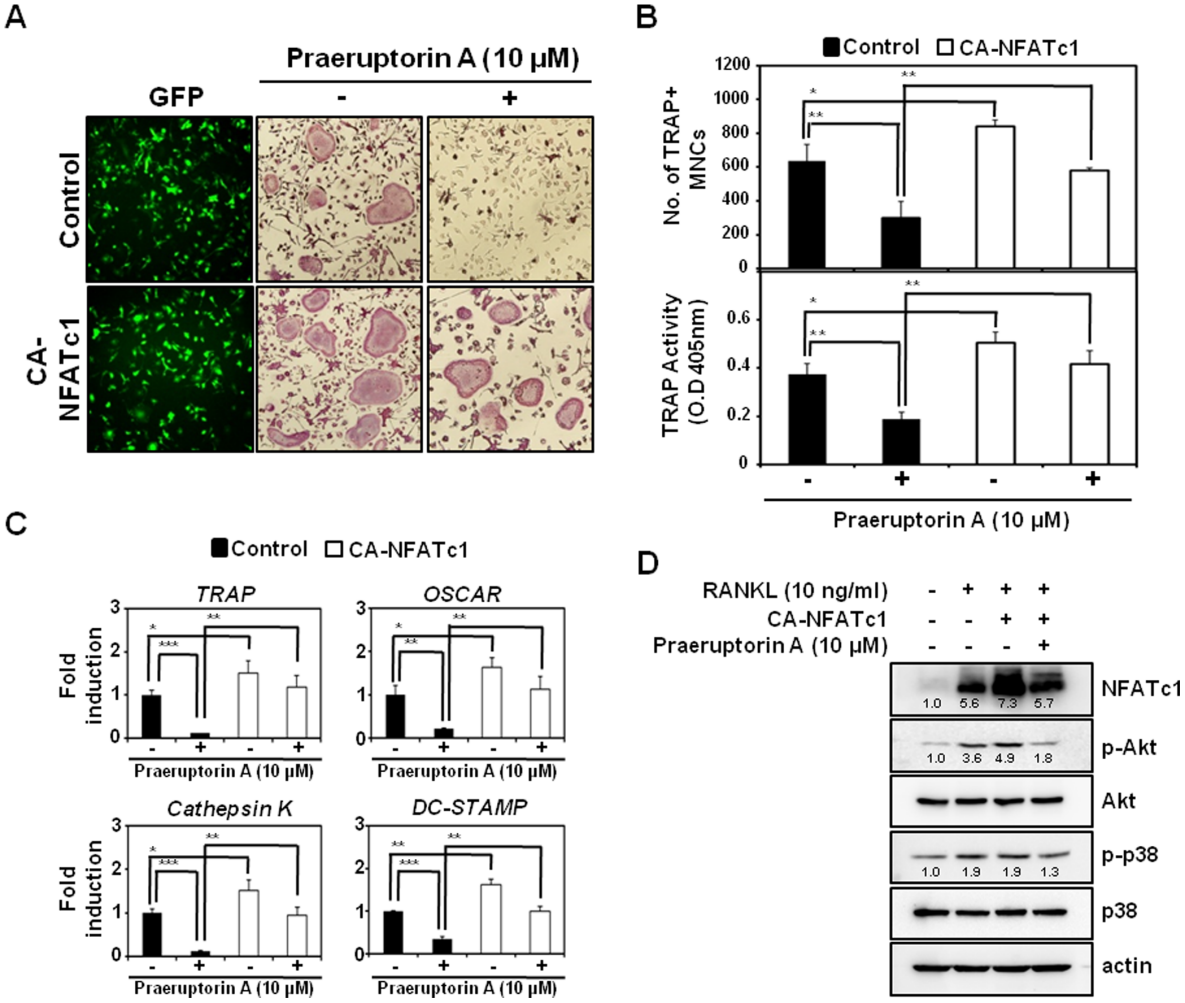
Effect of NFATc1 on anti-osteoclastogenic action of praeruptorin A. (A) BMMs were infected with retroviruses harboring the control GFP or Ca-NFATc1-GFP vectors. Transduced BMMs were cultured with RANKL (10 ng/ml) and M-CSF (30 ng/ml) in the presence of praeruptorin A (10 µM) or vehicle (DMSO). After incubation for 2 days, GFP expression was visualized under a fluorescence microscope. After 2 additional days, mature TRAP-positive multinucleated osteoclasts were visualized by TRAP staining. (B) TRAP-positive cells (nuclear number >3) were counted as osteoclasts, and TRAP activity was measured at 405 nm. On the differentiation day 2, the mRNA and protein expression levels of osteoclastogenesis-related molecules were analyzed by real-time PCR (C) and Western blot analysis, respectively (D). Densitometric analysis was performed using ImageJ software and the relative, normalized ratios of NFATc1/actin, p-Akt/Akt or p-p38/p38 were presented. *, *P*<0.05; **, *P*<0.01; ***P<0.001.

The anti-osteoclastogenic action of praeruptorin A could result from its potential to block p38 and/or Akt signaling pathways that subsequently affect the expression and/or activity of c-Fos and the most distal transcription factor, NFATc1. To clarify this hypothesis, we investigated whether ectopic expression of the constitutively active form of NFATc1 (CA-NFATc1) could rescue the praeruptorin A-inhibited formation of TRAP-positive multinucleated osteoclasts. Based on GFP signaling, both the control GFP and CA-NFATc1-GFP plasmid were infected well, and the overexpression of NFATc1 was confirmed by Western blot analysis (Fig. 4D). Consistent with Fig. 1B, the formation of TRAP-positive multinucleated osteoclasts from BMM expressing the control GFP was strongly inhibited by praeruptorin A (upper images in Fig. 4A). However, even in the presence of praeruptorin A, TRAP-positive multinucleated osteoclasts were derived from BMMs over-expressing NFATc1 (bottom images in Fig. 4A). The ameliorating effect of NFATc1 on the praeruptorin A-mediated inhibition of osteoclast differentiation was also confirmed by counting the number of multinucleated osteoclasts, measuring the TRAP activity (Fig. 4B), and evaluating the mRNA expression levels of TRAP, OSCAR, cathepsin K and DC-STAMP (Fig. 4C). Additionally, Western blot analysis revealed that praeruptorin A strongly attenuated the Akt activation by the overexpression of activated NFATc1 (Fig. 4D). The overexpression of activated NFATc1 did not induce the phosphorylation of p38.

### Praeruptorin A Inhibits Ca^2+^ Oscillation without Any Change in PLCγ Phosphorylation

**Figure 5:**
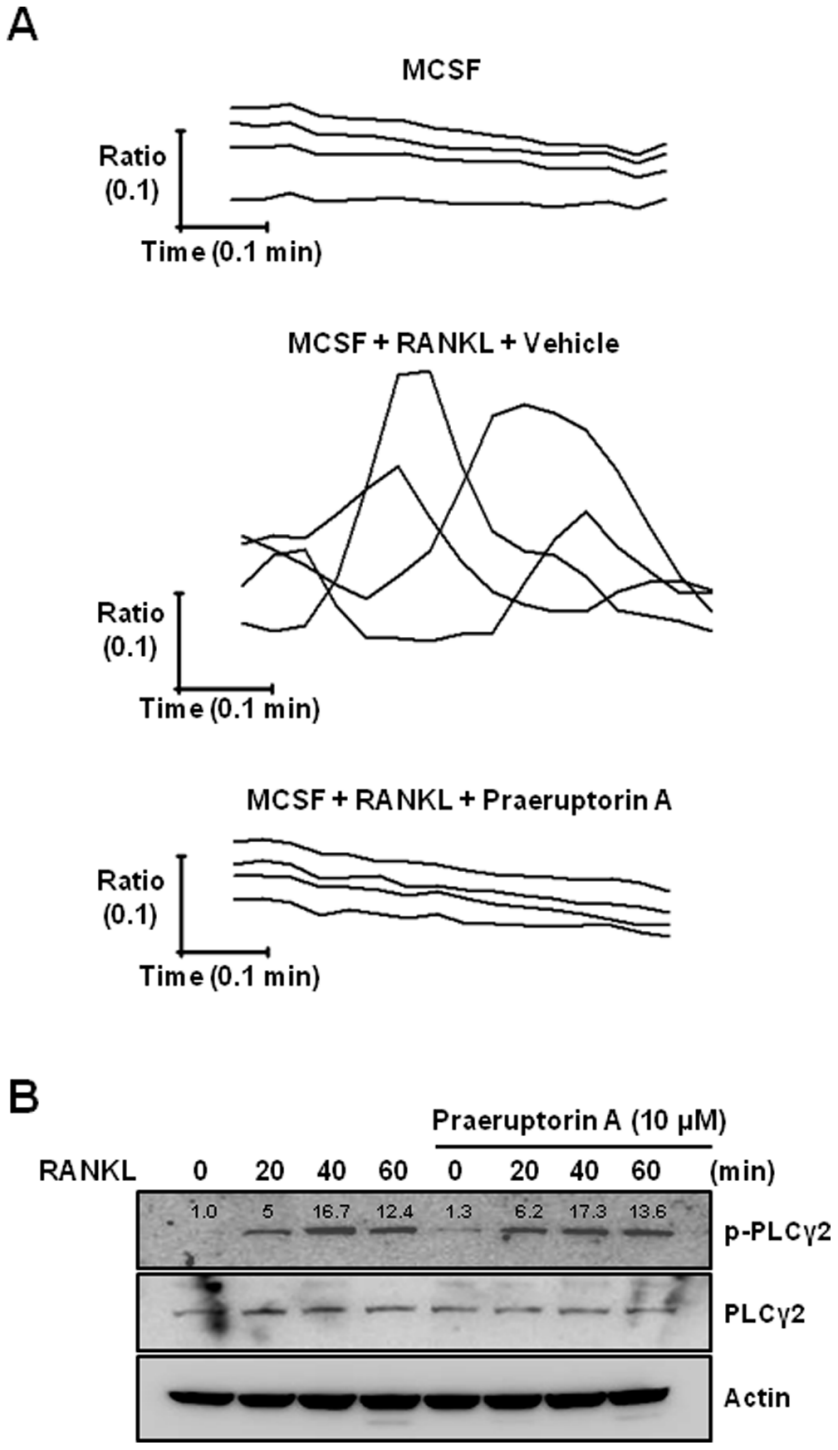
Effect of praeruptorin A on RANKL-induced Ca^2+^ oscillation and PLCγ phosphorylation. (A) The effect of praeruptorin A on the RANKL-induced Ca^2+^ oscillation was evaluated as described in ‘Materials and Methods’. Each trace presents intracellular Ca^2+^ mobilization in each cell. (B) The effect of praeruptorin A on the RANKL-induced phosphorylation of PLCγ was evaluated by Western blot analysis. BMMs were pre-treated with praeruptorin A for 2 h before treatment with RANKL. Actin was used as an internal control. Densitometric analysis was performed using ImageJ software and the relative, normalized ratio of p-PLCγ2/PLCγ2 was presented.

Since several studies have reported the Ca^2+^ channel blocking activity of praeruptorin A [23], [24], we further evaluated the effect of praeruptorin A on the RANKL-induced Ca^2+^ oscillation. As shown in Fig. 5A, when BMMs were treated with M-CSF plus RANKL for 24 h, Ca^2+^ oscillation was triggered, but not with M-CSF alone. However, additional treatment with praeruptorin A for 2 h before measuring Ca^2+^ oscillation completely inhibited the RANKL-induced Ca^2+^ oscillation.

We further examined the possibility that praeruptorin A could inhibit the RANKL-induced Ca^2+^ oscillation by blocking the RANKL-induced phosphorylation of PLCγ. In BMMs, the RANKL-induced phosphorylation of PLCγ was not changed by praeruptorin A.

## Discussion

Osteoclasts are functionally essential for sustaining bone health. However, the overactivation of osteoclasts and/or their increased number can lead to diseases characterized to bone loss, which is a risk factor for fracture. In this study, praeruptorin A attenuated the RANKL-induced osteoclastogenesis in a dose-dependent manner without any cytotoxicity upto 10 µM. Anti-osteoclastogenic activity of praeruptorin A was independent on age, strain and sex of mouse (Fig. S4), and importantly, praeruptorin A significantly inhibited the fusion of preosteoclasts (Fig. S2) and the formation of resorptive pits by mature multinucleated osteoclasts (Fig. S5).

RANKL is the most essential cytokine for osteoclast differentiation (or osteoclastogenesis) [25]. The binding of RANKL to RANK, its receptor, triggers the activation of signaling molecules such as MAP kinases, Akt, and phospholipase Cγ (PLCγ) that subsequently induce the activation of transcription factors such as c-Fos and nuclear factor of activated T cells (NFATc1) to regulate the expression of genes required for osteoclast differentiation [26]–[28]. c-Fos is an essential factor for the induction of NFATc1, which is a master transcription factor that regulates the process of osteoclast differentiation by controlling osteoclast-specific genes [29]–[31].

Here, praeruptorin A attenuated the RANKL-induced phosphorylation of p38 without affecting JNK and ERK. A pharmacological inhibition experiment using the p38 inhibitor SB203580 revealed direct involvement of p38 in the RANKL-induced osteoclast differentiation [22], [32], [33]. Furthermore, a study using both p38 inhibitor SB203580 and over-expression of dominant negative MKK3 and MKK6, which are known as upstream kinases of p38, revealed that the p38 signaling pathway could mediate the induction of c-Fos and NFATc1 during RANKL-stimulated osteoclast differentiation [34].

Moreover, praeruptorin A also attenuated the RANKL-induced phosphorylation of Akt. Akt has been known to play a critical role in the survival of osteoclasts rather than in osteoclast differentiation through the phosphoinositide 3-kinase (PI3K) kinase signaling pathway [35], [36]. However, a recent study showed the importance of the Akt-NFATc1 signaling axis in osteoclast differentiation [37]; inhibition of Akt phosphorylation by LY294002 resulted in the inhibition of osteoclast differentiation via modulation of RANKL-induced activation of NFATc1.

Thus, the anti-osteoclastogenic action of praeruptorin A could be due to its potential to inhibit both p38 and Akt signaling pathways that consequently downregulate the expression and/or activity of c-Fos and NFATc1. In particular, NFATc1, which is mainly regulated by c-Fos during osteoclastogenesis, plays a role as the most distal transcription factor required for regulating the expression of osteoclast-specific genes including TRAP, OSCAR, DC-STAMP, cathepsin K and c-Src [27], [28], [38]. TRAP is recognized as a marker of osteoclast differentiation and exhibits bone resorptive activity in the lysosomes [39]. OSCAR is a receptor that controls the PLCγ- Ca^2+^ signaling pathway, which is crucial for the activation of NFATc1 [40]. DC-STAMP and cathepsin K are well-known molecules for fusion and bone resorptive activity, respectively [38], [41]. c-Src tyrosine kinase is also required for the maintenance of the osteoclast actin cytoskeleton and the control of bone resorption [42]. In our results, praeruptorin A significantly inhibited the RANKL-induced expression of c-Fos, NFATc1 and those osteoclast-specific genes. Additionally, praeruptorin A inhibited the RANKL-induced activation of NFATc1.

These results suggested that the downregulation of NFATc1 could be the outcome of the anti-osteoclastogenic action of praeruptorin A by inhibiting p38 and Akt signaling pathways. The hypothesis was proved by the ectopic expression of the constitutively active form of NFATc1; it significantly rescued the anti-osteoclastogenic action of praeruptorin A. The rescue of defected osteoclastogenesis by overexpression of NFATc1 has been reported in several studies [9], [43]; for example, NFATc1-deficient embryonic stem cells failed to differentiate into osteoclasts after RANKL treatment, but the ectopic expression of NFATc1 rescued the abrogated osteoclast differentiation [30], [44]. Additionally, praeruptorin A strongly attenuated the Akt activation by the overexpression of activated NFATc1, but that did not induce the phosphorylation of p38. These results suggested that the autoamplification of NFATc1 during osteoclast differentiation could affect the activation of Akt, but not p38, and praeruptorin A has the potential to attenuate the NFATc1-mediated activation of Akt.

Furthermore, the overexpression of c-Fos did not significantly rescue the effect of praeruptorin A on osteoclast differentiation (Fig. S6), but the Western blot analysis revealed the involvement of NF-κB signaling in anti-osteoclastogenic action of praeruptorin A (Fig. S7). For degradation, IκBα was phosphorylated 5 min after RANKL treatment, and then free NF-κB p65 was translocated into nucleus in 15 to 30 min, but this activation of NF-κB by RANKL was shown to be attenuated by the pretreatment of praeruptorin A. The role of NF-κB in osteoclast differentiation has been described in several review articles [4], [5].

In osteoclast differentiation, RANKL also triggers the activation of PLCγ, which subsequently leads to Ca^2+^ mobilization [45]. As well as activating MAP kinases and Akt, PLCγ-medicated Ca^2+^ mobilization affects the activation of NFATc1 required for regulating osteoclast-specific genes [27]. Importantly, several studies have reported the Ca^2+^ channel blocking activity of praeruptorin A [23], [24]. These data clarify the hypothesis that praeruptorin A-mediated inhibition of Ca^2+^ oscillation via PLCγ could also downregulate the activity of NFATc1 during osteoclast differentiation. Interestingly, the RANKL-triggered Ca^2+^ oscillation was inhibited by praeruptorin A, but the RANKL-induced phosphorylation of PLCγ was not changed by praeruptorin A. These data suggest that the anti-osteoclastogenic activity of praeruptorin A involves inhibition of PLCγ-independent Ca^2+^ oscillation.

This is the first report of the anti-osteoclastogenic activity of praeruptorin A and its mode of action; praeruptorin A could inhibit the RANKL-induced osteoclast differentiation by inhibiting p38 and Akt signaling pathways and PLCγ-independent Ca^2+^ oscillation that consequently affect the expression and/or activity of the osteoclast-specific transcription factors, c-Fos and NFATc1. Also, NF-κB signaling was shown to be partly involved in the anti-osteoclastogenic action of praeruptorin A. In a further study, the binding molecules (or target proteins) of praeruptorin A might be identified, and the mechanism how praeruptorin A inhibits the fusion of preosteoclasts and the pit formation of mature osteoclasts might be elucidated.

## Supporting Information

Figure S1 **Effect of praeruptorin A on cell spreading during RANKL-induced osteoclast differentiation.** BMMs (1×10^4^ cells/well) were seeded in a 96-well plate, treated with the vehicle (0.1% DMSO) or praeruptorin A (10 µM) for 2 h in the presence of M-CSF (30 ng/ml), and incubated with RANKL (10 ng/ml) for 1 and 3 days. Then, cells were fixed, permeabilized, washed, and incubated with 10 µg/ml Hoechst 33342.
(TIF)

Figure S2 **Effect of praeruptorin A on RANKL-induced osteoclast differentiation for the indicated periods.** BMMs were cultured with praeruptorin A (10 µM) for various times periods (indicated the black arrow) in the presence of M-CSF and RANKL. After TRAP staining, TRAP-positive multinuclear cells (MNCs; nuclear number >3) were counted *, *P*<0.05; **, *P*<0.01; ***P<0.001.
(TIF)

Figure S3 **Effect of praeruptorin A on the formation of actin rings during osteoclast differentiation.** BMMs (1×10^4^ cells/well) were seeded in a 96-well plate, treated with the vehicle (0.1% DMSO) or praeruptorin A (10 µM) for 2 h in the presence of M-CSF (30 ng/ml), and incubated with RANKL (10 ng/ml) for 4 days. Then, cells were fixed, permeabilized, washed, and stained with Hoechst 33342 and phalloidin-FITC for nucleus and actin rings, respectively.
(TIF)

Figure S4 (A) Effect of age, strain or sex on anti-osteoclastogenic action of praeruptorin A. BMMs were isolated from mice (male and female ICR strain, 5-week old; male and female C57BL6/N strain, 5-week old and 8-month old) and cultured with praeruptorin A in the presence of M-CSF and RANKL for 4 days. Osteoclast differentiation was visualized by TRAP staining. (B) Effect of age, strain or sex on anti-osteoclastogenic action of praeruptorin A. BMMs were isolated from mice (male and female ICR strain, 5-week old; male and female C57BL6/N strain, 5-week old and 8-month old) and cultured with praeruptorin A in the presence of M-CSF and RANKL for 4 days. After TRAP staining, TRAP-positive multinuclear cells (MNCs; nuclear number >3) were counted. TRAP activity and cell viability were also evaluated. *, *P*<0.05; **, *P*<0.01; ***P<0.001.
(TIF)

Figure S5 **Anti-resorptive activity of praeruptorin A.** (A) After co-culturing BMMs with osteoblasts for 7 days, multinucleated osteoclasts were replated on BioCoat Osteologic MultiTest slides and after 2 h incubation, cells were further incubated with praeruptorin A and RANKL for 6 h. Then, cells were stained for TRAP (upper images). (B) TRAP-positive multinucleated cells were counted. (C) After removing cells, the resorption pits (indicated by asterisks in bottom images) were observed under a light microscope. The relative resorbing areas were evaluated using the ImageJ program. ***P<0.001.
(TIF)

Figure S6 **Effect of c-Fos on anti-osteoclastogenic action of praeruptorin A.** BMMs were infected with retroviruses harboring the control GFP or c-Fos-GFP vectors. Transduced BMMs were cultured with RANKL (10 ng/ml) and M-CSF (30 ng/ml) in the presence of praeruptorin A (10 µM) or the vehicle (0.1% DMSO). (A) After incubation for 2 days, GFP expression was visualized under a fluorescence microscope (left images). After 2 additional days, mature TRAP-positive multinucleated osteoclasts were visualized by TRAP staining (middle and right images). TRAP-positive cells (nuclear number >3) were counted as osteoclasts (B), and TRAP activity was measured at 405 nm (C). *, *P*<0.05; **, *P*<0.01; ***P<0.001.
(TIF)

Figure S7 **Effect of praeruptorin A on RANKL-induced activation of NF-κB signaling pathway.** BMMs were treated with praeruptorin A (10 µM) for 30 min, stimulated with RANKL (10 ng/ml) for the indicated time. The expression levels of molecules in cytoplasmic or nuclear protein fractions were evaluated by Western blot analysis. Actin and lamin B1 were used for the loading control of cytosolic and nuclear proteins, respectively. Densitometric analysis was performed using ImageJ software and the relative, normalized ratios of IκBα/actin, p-IκBα/actin, cytosolic p65/actin or nuclear p65/lamin B1 were presented.
(TIF)

File S1 **Materials and Methods.**
(DOC)
