# Supplementary figures and images for: Anti-Osteoclastogenic Activity of Praeruptorin A via Inhibition of p38/Akt-c-Fos-NFATc1 Signaling and PLCγ-Independent Ca2+ Oscillation
Source: PLoS One. 2014 Feb 21;9(2):e88974. doi: 10.1371/journal.pone.0088974 (PMC3931687; doi:10.1371/journal.pone.0088974)

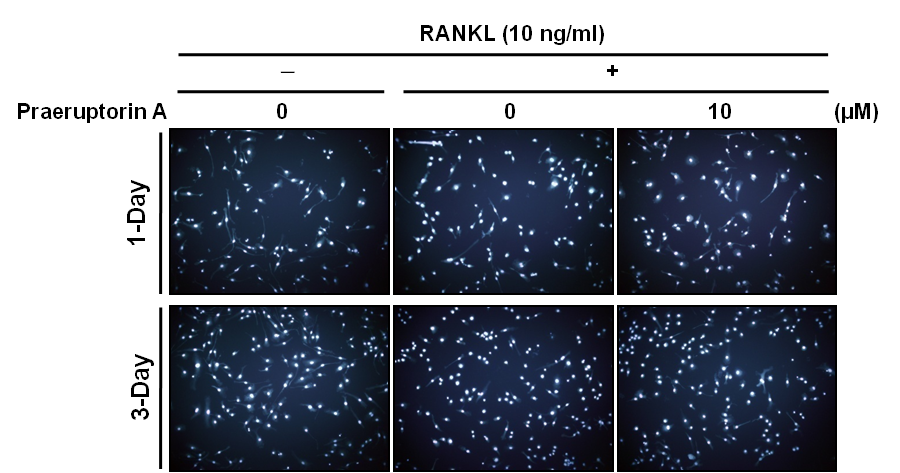

Supplement: Figure S1 — Effect of praeruptorin A on cell spreading during RANKL-induced osteoclast differentiation. BMMs (1×10^4^ cells/well) were seeded in a 96-well plate, treated with the vehicle (0.1% DMSO) or praeruptorin A (10 µM) for 2 h in the presence of M-CSF (30 ng/ml), and incubated with RANKL (10 ng/ml) for 1 and 3 days. Then, cells were fixed, permeabilized, washed, and incubated with 10 µg/ml Hoechst 33342. (TIF) [file pone.0088974.s001.tif]

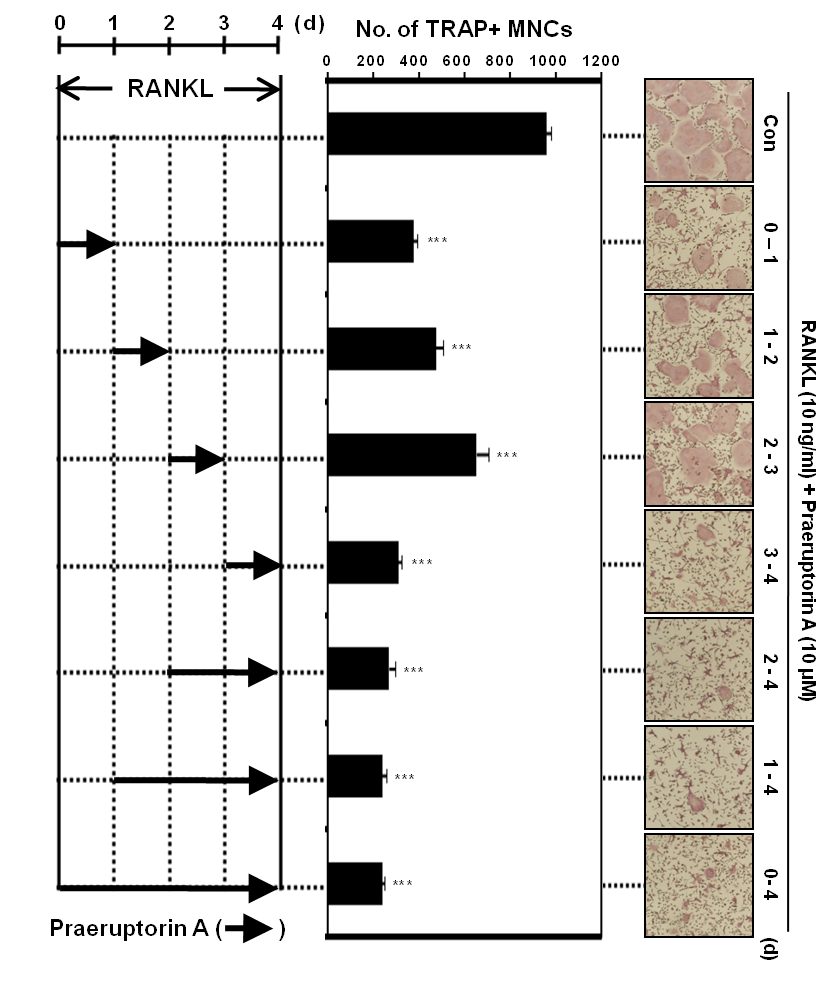

Supplement: Figure S2 — Effect of praeruptorin A on RANKL-induced osteoclast differentiation for the indicated periods. BMMs were cultured with praeruptorin A (10 µM) for various times periods (indicated the black arrow) in the presence of M-CSF and RANKL. After TRAP staining, TRAP-positive multinuclear cells (MNCs; nuclear number >3) were counted *, P<0.05; **, P<0.01; ***P<0.001. (TIF) [file pone.0088974.s002.tif]

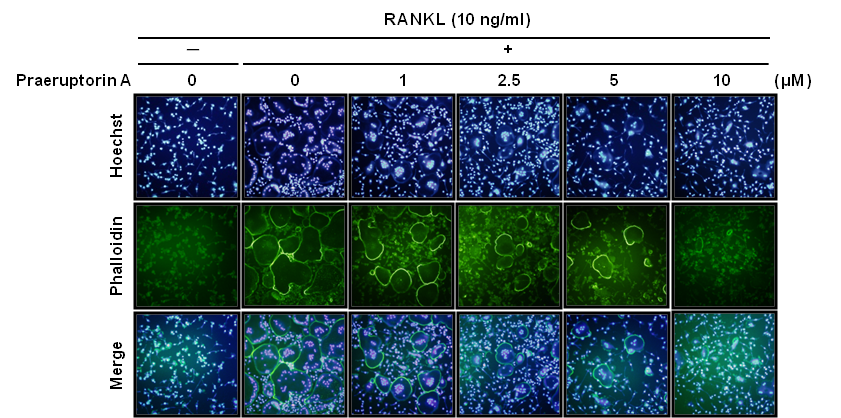

Supplement: Figure S3 — Effect of praeruptorin A on the formation of actin rings during osteoclast differentiation. BMMs (1×10^4^ cells/well) were seeded in a 96-well plate, treated with the vehicle (0.1% DMSO) or praeruptorin A (10 µM) for 2 h in the presence of M-CSF (30 ng/ml), and incubated with RANKL (10 ng/ml) for 4 days. Then, cells were fixed, permeabilized, washed, and stained with Hoechst 33342 and phalloidin-FITC for nucleus and actin rings, respectively. (TIF) [file pone.0088974.s003.tif]

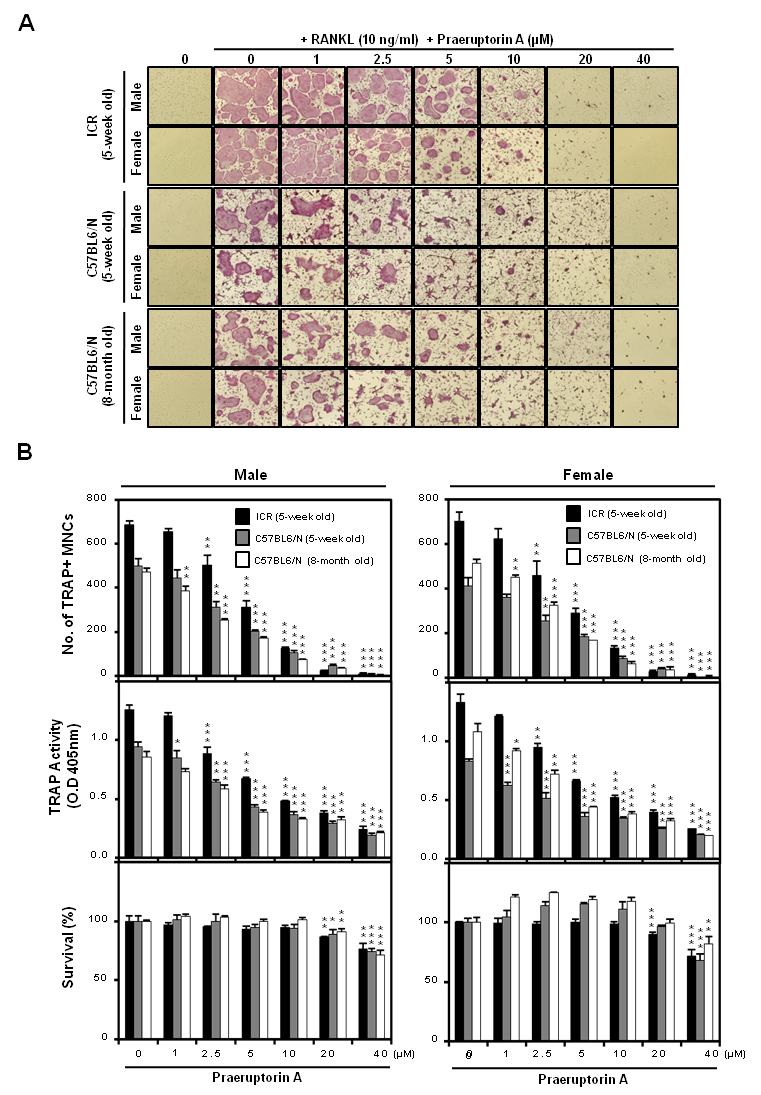

Supplement: Figure S4 — (A) Effect of age, strain or sex on anti-osteoclastogenic action of praeruptorin A. BMMs were isolated from mice (male and female ICR strain, 5-week old; male and female C57BL6/N strain, 5-week old and 8-month old) and cultured with praeruptorin A in the presence of M-CSF and RANKL for 4 days. Osteoclast differentiation was visualized by TRAP staining. (B) Effect of age, strain or sex on anti-osteoclastogenic action of praeruptorin A. BMMs were isolated from mice (male and female ICR strain, 5-week old; male and female C57BL6/N strain, 5-week old and 8-month old) and cultured with praeruptorin A in the presence of M-CSF and RANKL for 4 days. After TRAP staining, TRAP-positive multinuclear cells (MNCs; nuclear number >3) were counted. TRAP activity and cell viability were also evaluated. *, P<0.05; **, P<0.01; ***P<0.001. (TIF) [file pone.0088974.s004.tif]

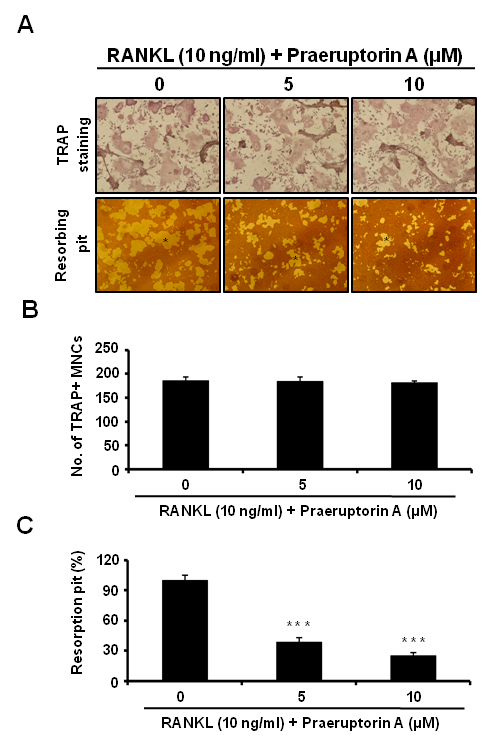

Supplement: Figure S5 — Anti-resorptive activity of praeruptorin A. (A) After co-culturing BMMs with osteoblasts for 7 days, multinucleated osteoclasts were replated on BioCoat Osteologic MultiTest slides and after 2 h incubation, cells were further incubated with praeruptorin A and RANKL for 6 h. Then, cells were stained for TRAP (upper images). (B) TRAP-positive multinucleated cells were counted. (C) After removing cells, the resorption pits (indicated by asterisks in bottom images) were observed under a light microscope. The relative resorbing areas were evaluated using the ImageJ program. ***P<0.001. (TIF) [file pone.0088974.s005.tif]

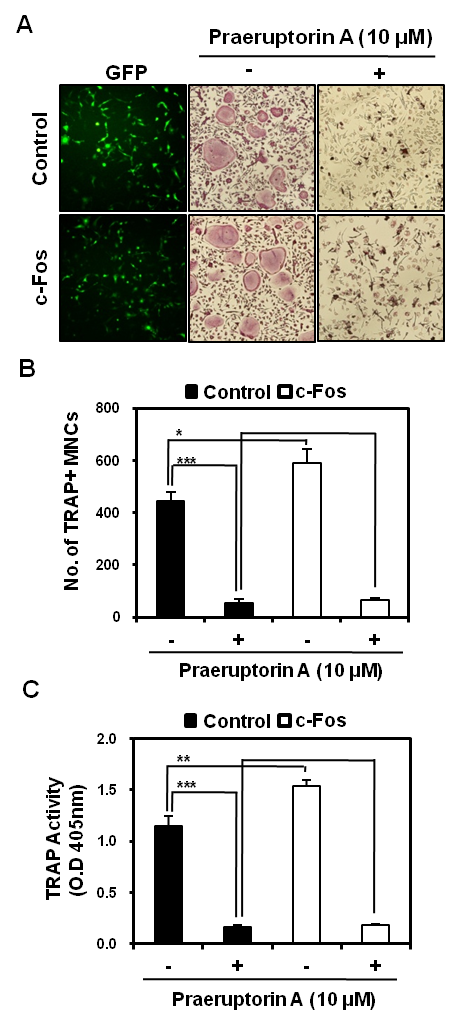

Supplement: Figure S6 — Effect of c-Fos on anti-osteoclastogenic action of praeruptorin A. BMMs were infected with retroviruses harboring the control GFP or c-Fos-GFP vectors. Transduced BMMs were cultured with RANKL (10 ng/ml) and M-CSF (30 ng/ml) in the presence of praeruptorin A (10 µM) or the vehicle (0.1% DMSO). (A) After incubation for 2 days, GFP expression was visualized under a fluorescence microscope (left images). After 2 additional days, mature TRAP-positive multinucleated osteoclasts were visualized by TRAP staining (middle and right images). TRAP-positive cells (nuclear number >3) were counted as osteoclasts (B), and TRAP activity was measured at 405 nm (C). *, P<0.05; **, P<0.01; ***P<0.001. (TIF) [file pone.0088974.s006.tif]
