# Supplementary material for: Anti-Osteoclastogenic Activity of Praeruptorin A via Inhibition of p38/Akt-c-Fos-NFATc1 Signaling and PLCγ-Independent Ca2+ Oscillation
Source: PLoS One. 2014 Feb 21;9(2):e88974. doi: 10.1371/journal.pone.0088974 (PMC3931687; doi:10.1371/journal.pone.0088974)
Supplement: File S1 — Materials and Methods. (DOC) [file pone.0088974.s008.doc]

**Supporting Information**

***Hoechst staining***

BMMs (1 × 10^4^ cells/well) were seeded in a 96-well plate, treated with the vehicle (0.1% DMSO) or praeruptorin A (10 μM) for 2 h in the presence of M-CSF (30 ng/ml), and incubated with RANKL (10 ng/ml) for 1 and 3 days. Then, cells were fixed with 10% formalin for 5 min, permeabilized with 0.1% Triton X-100 in PBS for 5 min, washed twice with PBS, and incubated with 10 μg/ml Hoechst 33342 (Carlsbad, CA) for 5 min. Images of stained nucleus were captured under a fluorescent microscope with a DP Controller.

***Osteoclast differentiation***

To evaluate the stage-dependent anti-osteoclastogenic activity of praeruptorin A, BMMs were isolated and cultured with praeruptorin A (10 μM) for various times periods in the presence of M-CSF and RANKL for osteoclast differentiation. After TRAP staining, TRAP-positive multinuclear cells with three or more nuclei were counted as osteoclasts. Furthermore, in order to investigate whether age, strain or sex could affect anti-osteoclastogenic activity of praeruptorin A, mice (male and female ICR strain, 5-week old; male and female C57BL6/N strain, 5-week old and 8-month old) were purchased from Central Lab. Animal Inc. (Seoul, Korea), and as described in the ‘Materials and Methods’, BMMs were isolated and cultured with praeruptorin A in the presence of M-CSF and RANKL for osteoclast differentiation.

***Actin ring staining***

On the differentiation day 4, cells were fixed with 10% formalin for 5 min, permeabilized with 0.1% Triton X-100 in PBS for 5 min, and washed with PBS. Actin rings were stained with 50 μg/ml phalloidin-FITC (Sigma-Aldrich, MO) for 30 min under the condition of light protection. After washing twice with PBS, nuclei were stained with 10 μg/ml Hoechst 33342 for 5 min. Images of stained nucleus were captured under a fluorescent microscope with a DP Controller.

***Bone pit formation analysis***

BMMs and osteoblasts isolated from the calvariae of newborn mice by serial digestion with collagenase were co-cultured with 1α, 25(OH)_2_D_3_ (10^-8^ M) and PGE2 (10^-6^ M) in the collagen-coated dishes. After 7 days, multinucleated osteoclasts were replated on BioCoat Osteologic MultiTest slides and after 2 h incubation, cells were further incubated with praeruptorin A and RANKL for 6 h. Then, cells were stained for TRAP, and photographs were taken under a light microscope at 40 × magnification. To observe resorption pits, cells on the BioCoat slides were washed with PBS and treated with 5% sodium hypochlorite for 5 min. After washing the plate with PBS buffer and drying, the resorption pits were observed under a light microscope. Quantification of resorbed areas was performed using the ImageJ program.

***Retrovirus preparation and infection***

Retrovirus preparation and infection were conducted as described in ‘Materials and Methods’. Briefly, to obtain retroviral particles, plasmids of pMX-IRES-GFP (the control) and pMX-c-Fos-GFP were transfected into Plat-E cells using Lipofectamine 2000 reagent. After viral particles were collected from the culture medium for 48 h, BMMs were incubated with those in the presence of M-CSF (30 ng/ml) and polybrene (10 μg/mL) for 8 h. For the osteoclast formation assay, BMMs were treated with RANKL (10 ng/ml), M-CSF (30 ng/ml) and praeruptorin A (10 μM) for 4 days.

***Western blot analysis***

Cytoplasmic or nuclear protein fractions were prepared using a NucBuster Protein Extraction kit (Novagen, Germany). After protein quantification, Western blot analysis was performed as described in ‘Material and Methods’. Antibodies against p-IκBα, IκBα and NF-κB/p65 were purchased from Santa Cruz Biotechnology (CA). Antibody against Lamin B1 was obtained from AbFrontier (Seoul, Korea). Lamin B1 was used for the loading control of nuclear proteins.

***Statistical analysis***

All quantitative values are presented as mean ± SD. Statistical differences were analyzed using Student’s *t-*test. A value of *p* < 0.05 was considered significant.

**Supporting Information Legends**

**Figure S1.** Effect of praeruptorin A on cell spreading during RANKL-induced osteoclast differentiation. BMMs (1 × 10^4^ cells/well) were seeded in a 96-well plate, treated with the vehicle (0.1% DMSO) or praeruptorin A (10 μM) for 2 h in the presence of M-CSF (30 ng/ml), and incubated with RANKL (10 ng/ml) for 1 and 3 days. Then, cells were fixed, permeabilized, washed, and incubated with 10 μg/ml Hoechst 33342.

**Figure S2.** Effect of praeruptorin A on RANKL-induced osteoclast differentiation for the indicated periods. BMMs were cultured with praeruptorin A (10 μM) for various times periods (indicated the black arrow) in the presence of M-CSF and RANKL. After TRAP staining, TRAP-positive multinuclear cells (MNCs; nuclear number > 3) were counted *, *P* < 0.05; **, *P* < 0.01; *** *P* < 0.001.

**Figure S3.** Effect of praeruptorin A on the formation of actin rings during osteoclast differentiation. BMMs (1 × 10^4^ cells/well) were seeded in a 96-well plate, treated with the vehicle (0.1% DMSO) or praeruptorin A (10 μM) for 2 h in the presence of M-CSF (30 ng/ml), and incubated with RANKL (10 ng/ml) for 4 days. Then, cells were fixed, permeabilized, washed, and stained with Hoechst 33342 and phalloidin-FITC for nucleus and actin rings, respectively.

**Figure S4.** (A) Effect of age, strain or sex on anti-osteoclastogenic action of praeruptorin A. BMMs were isolated from mice (male and female ICR strain, 5-week old; male and female C57BL6/N strain, 5-week old and 8-month old) and cultured with praeruptorin A in the presence of M-CSF and RANKL for 4 days. Osteoclast differentiation was visualized by TRAP staining. (B) Effect of age, strain or sex on anti-osteoclastogenic action of praeruptorin A. BMMs were isolated from mice (male and female ICR strain, 5-week old; male and female C57BL6/N strain, 5-week old and 8-month old) and cultured with praeruptorin A in the presence of M-CSF and RANKL for 4 days. After TRAP staining, TRAP-positive multinuclear cells (MNCs; nuclear number > 3) were counted. TRAP activity and cell viability were also evaluated. *, *P* < 0.05; **, *P* < 0.01; *** *P* < 0.001.

**Figure S5.** Anti-resorptive activity of praeruptorin A. (A) After co-culturing BMMs with osteoblasts for 7 days, multinucleated osteoclasts were replated on BioCoat Osteologic MultiTest slides and after 2 h incubation, cells were further incubated with praeruptorin A and RANKL for 6 h. Then, cells were stained for TRAP (upper images). (B) TRAP-positive multinucleated cells were counted. (C) After removing cells, the resorption pits (indicated by asterisks in bottom images) were observed under a light microscope. The relative resorbing areas were evaluated using the ImageJ program. *** *P* < 0.001

**Figure S6.** Effect of c-Fos on anti-osteoclastogenic action of praeruptorin A. BMMs were infected with retroviruses harboring the control GFP or c-Fos-GFP vectors. Transduced BMMs were cultured with RANKL (10 ng/ml) and M-CSF (30 ng/ml) in the presence of praeruptorin A (10 μM) or the vehicle (0.1% DMSO). (A) After incubation for 2 days, GFP expression was visualized under a fluorescence microscope (left images). After 2 additional days, mature TRAP-positive multinucleated osteoclasts were visualized by TRAP staining (middle and right images). TRAP-positive cells (nuclear number > 3) were counted as osteoclasts (B), and TRAP activity was measured at 405 nm (C). *, *P* < 0.05; **, *P* < 0.01; *** *P* < 0.001

**Figure S7.** Effect of praeruptorin A on RANKL-induced activation of NF-κB signaling pathway. BMMs were treated with praeruptorin A (10 μM) for 30 min, stimulated with RANKL (10 ng/ml) for the indicated time. The expression levels of molecules in cytoplasmic or nuclear protein fractions were evaluated by Western blot analysis. Actin and lamin B1 were used for the loading control of cytosolic and nuclear proteins, respectively. Densitometric analysis was performed using ImageJ software and the relative, normalized ratios of IκBα/actin, p-IκBα/actin, cytosolic p65/actin or nuclear p65/lamin B1 were presented.
